# Development of Novel Intramolecular FRET-Based ABC Transporter Biosensors to Identify New Substrates and Modulators

**DOI:** 10.3390/pharmaceutics10040186

**Published:** 2018-10-13

**Authors:** Bremansu Osa-Andrews, Kee W. Tan, Angelina Sampson, Surtaj H. Iram

**Affiliations:** Department of Chemistry & Biochemistry, College of Arts and Sciences, South Dakota State University, Brookings, SD 57007, USA; bremansu@gmail.com (B.O.-A.); ktan097@gmail.com (K.W.T.); angelina.sampson@sdstate.edu (A.S.)

**Keywords:** ATP-binding cassette (ABC) proteins, multidrug resistance, fluorescence resonance energy transfer, biosensors, multidrug resistance protein 1, two-color MRP1

## Abstract

Multidrug resistance protein 1 (MRP1) can efflux a wide variety of molecules including toxic chemicals, drugs, and their derivatives out of cells. Substrates of MRP1 include anti-cancer agents, antibiotics, anti-virals, anti-human immunodeficiency virus (HIV), and many other drugs. To identify novel substrates and modulators of MRP1 by exploiting intramolecular fluorescence resonance energy transfer (FRET), we genetically engineered six different two-color MRP1 proteins by changing green fluorescent protein (GFP) insertion sites, while keeping the red fluorescent protein (RFP) at the C-terminal of MRP1. Four of six recombinant proteins showed normal expression, localization, and transport activity. We quantified intramolecular FRET using ensemble fluorescence spectroscopy in response to binding of known substrate or ATP alone, substrate/ATP, and trapping of the transporter in closed conformation by vanadate. Recombinant MRP1 proteins GR-881, GR-888, and GR-905 exhibited reproducible and higher FRET changes under all tested conditions and are very promising for use as MRP1 biosensors. Furthermore, we used GR-881 to screen 40 novel anti-cancer drugs and identified 10 hits that potentially directly interact with MRP1 and could be substrates or modulators. Profiling of drug libraries for interaction with MRP1 can provide very useful information to improve the efficacy and reduce the toxicity of various therapies.

## 1. Introduction

ATP-binding cassette (ABC) membrane proteins are a large superfamily of proteins consisting of seven subfamilies (A to G), which mediate the ATP-dependent transport of diverse solutes including lipids, peptides, heavy metals, ions, and a wide variety of endogenous and exogenous compounds and their metabolites across biological membranes [1,2,3,4,5,6,7]. Ubiquitously found in all phyla, ABC transporters are unidirectional importers or exporters in bacteria, but only exporters in eukaryotes [8,9]. When overexpressed in tumors, ABC proteins such as P-glycoprotein (P-gp/ABCB1), multidrug drug resistance protein 1 (MRP1/ABCC1), and breast cancer resistance protein (BCRP/ABCG2) are implicated in poor patient response to chemotherapy. These ABC transporters function in an ATP-dependent manner and efflux their substrate drugs out of cells, negatively impacting the efficacy of those drugs. MRP1 can also efflux a remarkable variety of xenobiotics and organic anions from endogenous sources, which are mostly conjugated to glutathione, glucuronide, or sulfate [10,11].

The prototypical functional ABC transporter is composed of four domains, two membrane spanning domains (MSDs), each containing six transmembrane (TM) α-helices, and two nucleotide-binding domains (NBDs) that are cytosolic [12,13,14]. The structure of MRP1 and several “C” subfamily members contains five domains. The two membrane-spanning domains (MSD1 and MSD2) [15,16] are intertwined to form the substrate-binding site/s and the substrate translocation pathway, and an additional MSD (MSD0) at the amino-terminus of the protein, whose specific biological function is poorly understood [9,17,18]. When bound with ATP and substrate, the two cytosolic NBDs dimerize in a head-to-tail orientation. The energy required to translocate the substrates is generated by the binding and hydrolysis of ATP [19,20,21].

Overexpression of MRP1 was reported to confer multidrug resistance in acute leukemia, prostate cancer, breast cancer, and neuroblastoma by actively pumping out anti-cancer agents, thereby preventing the accumulation of anti-cancer drugs, eventually causing poor chemotherapeutic outcomes [10,22,23,24]. In addition to anti-cancer agents, MRP1 can reduce the efficacy of a wide variety of drugs commonly used for various metabolic diseases and neurological disorders, as well as antivirals, antimalarials, antibiotics, antidepressants, and anti-human immunodeficiency virus (HIV) drugs [1,25,26]. The strategic distribution of human MRP1 at “pharmacological sanctuary sites” such as the blood–brain and blood–testis barriers, indicates the chemoprotective and tissue-defensive roles of the protein [10]. Consequently, MRP1 plays an important role in the absorption and disposition of a remarkably diverse set of substrates across different organs and physiological barriers [27]. The critical role MRP1 plays in health and disease is further corroborated by its involvement in biochemical processes such as redox homeostasis, cellular processes such as hormone secretion, and the etiology of neurodegenerative, immunological, and cardiovascular pathologies [28,29,30,31,32,33,34,35].

Generally, drug development involves painstaking steps which include basic research, in vitro drug screening, in vivo experimentation, preclinical stage, stage 1 to stage 4 clinical trials, expending billions of dollars before the drug can be accessible to the population [36]. However, a significant percentage of drugs fail in the clinical trials due to a lack of efficacy and toxicity issues, costing billions of dollars. Profiling drug–transporter interactions may allow discarding undesirable compounds at the very early stage to reduce economic burden and may avoid potential drug–drug or food–drug interactions. Furthermore, profiling drug interactions with MRP1 can identify drugs that are at high or low risk of developing multidrug resistance, as well as discovery of novel inhibitors useful for clinical chemotherapy, especially in cancers where MRP1 is overexpressed and contributes to multidrug resistance. 

**Figure 1:**
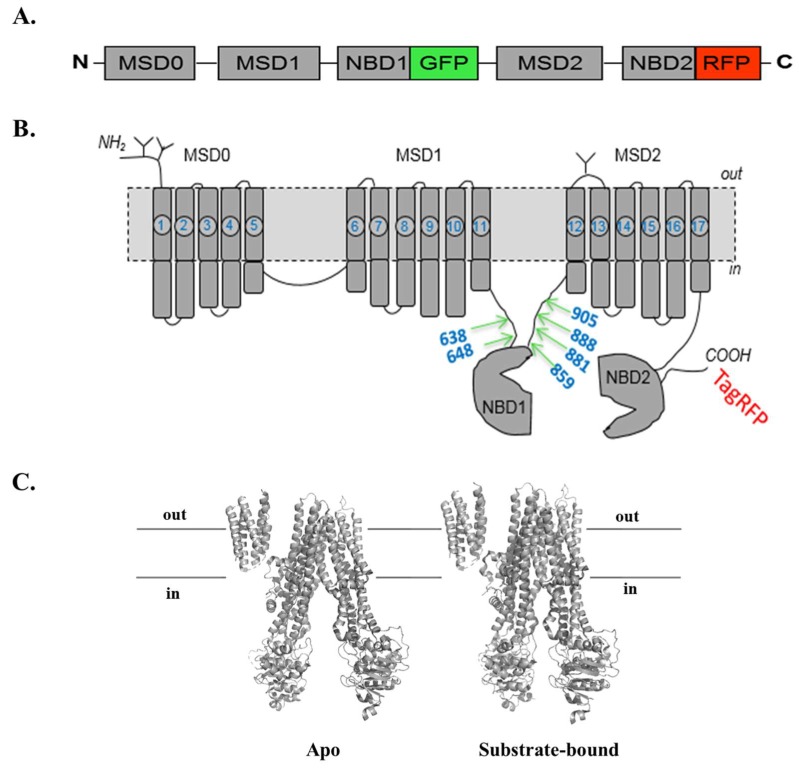
Schematic of two-color multidrug resistance protein 1 (MRP1) structures. (**A**) Schematic diagram of two-color MRP1 construct showing C-terminal red fluorescent protein (TagRFP) and an intra-sequence green fluorescent protein (GFP). (**B)** Predicted secondary structure of two-color MRP1 showing the three membrane-spanning domains (MSD0, MSD1, and MSD2), the two cytoplasmic nucleotide binding domains (NBD1 and NBD2), and the GFP insertion sites for engineering the constructs. The numbers 638, 648, 860, 881, 888, and 905 represent the amino-acid residue number of wild-type MRP1 after which GFP was inserted. TagRFP was fused to the C-terminal of MRP1. (**C)** Structure of bovine MRP1 in the apo (left, Protein Data Bank (PDB) code: 5UJ9) and substrate-bound (right, PDB code: 5UJA) conformations.

To investigate the structural changes of MRP1 during transport activity in live cells, we previously engineered a two-color MRP1 construct by inserting green fluorescent protein (GFP) and red fluorescent protein (TagRFP) at the C-terminus of NBD1 and NBD2, respectively (Figure 1A), and quantified intramolecular fluorescence resonance energy transfer (FRET) changes as an index of NBD conformational changes [26]. We also used the two-color MRP1 to detect substrate/inhibitor/activator candidates and identified eight compounds that directly interact and induce a conformational change in the structure of MRP1 after screening of the National Institutes of Health (NIH) Clinical Collection (NCC; a library of 446 drug-like compounds) [26]. Six of the eight hits were later found to directly interact with MRP1 [37], while further work is needed to verify the interaction of the remaining two hits. These findings validated the utility of the intramolecular FRET-based approach using two-color MRP1 to identify compounds that directly interact with MRP1. Our claim that substrate binding results in conformational changes in the NBDs and brings them closer was further supported by recent high-resolution structures of bovine MRP1 in apo and substrate-bound states (Figure 1C). However, based on our ongoing work and our expert knowledge of MRP1 substrate specificity, we expected many more hits from screening 446 NIH drug-like compounds. We strongly felt that many potential compounds that directly interact with MRP1 were missed out in this screening due to high noise-to-signal ratio. In our proof-of-concept study, we used a highly specialized fluorescence lifetime microplate reader for high-throughput drug screening, which is not yet commercially available through an established manufacturer. In addition, the FRET change signal upon ligand interaction was not sufficiently high to perform screening with a more commonly used fluorescence intensity microplate reader or a fluorometer. In order to make this innovative and potentially transformative technology more readily accessible, economical, and commercially more attractive, we decided to create a more efficient two-color MRP1 construct, which can produce a bigger FRET change signal upon interaction with the substrate. Therefore, our objective in this study was to engineer several new two-color MRP1 recombinant proteins by altering the GFP insertion site and to identify the most efficient FRET reporter of NBD conformational changes in MRP1. 

## 2. Materials and Methods

### 2.1. Chemicals

Nucleotides, doxorubicin, anti-GFP antibody, benzamidine, poly-d-lysine, saponin, and 2-mercaptoethanol were purchased from Sigma Aldrich (St. Louis, MO, USA). Estradiol glucuronide (E217βG) and sodium orthovanadate were obtained from Santa Cruz (Dallas, TX, USA). MK571 was received from Cayman Chemicals (Ann Arbor, MI, USA). Restriction enzymes were purchased from New England Biolabs (New England, MA, USA) and phosphate-buffered saline (PBS) was purchased from Thermo Scientific (Sunnyvale, CA, USA).

### 2.2. Engineering Two-Color Multidrug Resistance Protein 1 (MRP1) Constructs

All the genetically engineered two-color MRP1 proteins have a pTagRFP-N vector as a backbone. To engineer the GR-638 construct, firstly, a C-terminal 2.6-kb insert fragment of MRP1 which encodes amino acids 639–1531 (stop codon removed) was generated by polymerase chain reaction (PCR) using the following primers: forward, 5’ GTA CCG CGG GGG GGC ACG AAC AGC ATC ACC G 3’; reverse, 5’ GTA ACC GGT CT CAC CAA GCC GGC GTC TTT GGC CAT G 3’. It was cloned in frame with TagRFP (underlined sequence is the designed restriction site). Secondly, a 0.7-kb insert fragment which codes for GFP was amplified using the following primers: forward, 5’ GTA GTC GAC ATG GTG AGCAAG GGC GAG GAG 3’; reverse, 5’ GTA CCG CGG CTT GTA CAG CTC GTC CAT GCC 3’. It was fused to the construct. In the third step, an N-terminal 2-kb fragment of MRP1, which codes for amino acids 1–638, was amplified with the following primers: forward, 5’ GTA GAG CTC ATG GCG CTC CGG GGC TTC TGC AG 3’; reverse, 5’ CTA GTC GAC GCC GTC TTT GAC AGG CCG TCG CTC 3’. It was inserted in frame with GFP to generate the final construct. The GFP sequence in the final two-color recombinant protein construct was separated from the N-terminus and C-terminus of MRP1 via amino-acid linkers, valine–aspartate and proline–arginine, respectively. With the stop codon of MRP1 eliminated, the C-terminal end of the protein was ligated to TagRFP, separated by a linker made of five amino acids—threonine, glycine, leucine, alanine, and threonine. 

The schematic diagram of the six engineered two-color MRP1s is shown in Figure 1A. A similar cloning strategy was employed to engineer five other constructs distinguishable by the variants of GFP insertion sites. Accordingly, constructs GR-648, GR-859, GR-881, GR-888, and GR-905 were cloned by inserting GFP after amino-acid residues 648, 859, 881, 888, and 905, respectively, in the MRP1 protein. The primer sequences of GFP, as well as those of N-terminal and C-terminal MRP1 fragments, are shown in Appendix B. A schematic diagram of two-color MRP1, showing the distinctive GFP insertion sites, is shown in Figure 1B. A tertiary structure representation of bovine MRP1 in open (substrate-free) and closed (substrate-bound) conformations is shown in Figure 1C.

### 2.3. Cell Lines and Cell Culture

The HEK293T (human embryonic kidney) cell line was generously donated by Dr. Adam Hoppe (South Dakota State University, Brookings, SD, USA). Dulbecco’s modified Eagle medium (DMEM) (GE Healthcare, Marlborough, MA, USA) enriched with 10% fetal bovine serum (FBS) was used to grow the HEK293 cell lines. Cell lines were grown in a humidified incubator maintained at 5% CO_2_ and 37 °C. This incubation condition was retained in all subsequent cell culture procedures.

### 2.4. Preparation of MRP1-Enriched Membrane Vesicles

Membrane vesicles were prepared based on the method described by Leo et al. (1996) with minor alterations. Thawed frozen cell pellets from −80 °C were resuspended in homogenization buffer (250 mM sucrose, 50 mM Tris-HCl, and 0.25 mM CaCl_2_) supplemented with 1× protease inhibitor cocktail (enriched with 1mM ethylenediaminetetraacetic acid (EDTA)). Cell suspension was pressurized to 450 psi in a pre-cooled nitrogen bomb chamber for 5 min to disrupt the plasma membrane. Exploded cell lysates were then centrifuged at 500× *g* at 4 °C for 10 min. The supernatant was collected into clean pre-cooled high-speed ultracentrifuge tubes, while the cell pellets were re-suspended in homogenization buffer and re-centrifuged to pool the second supernatant. The entire supernatant retrieved was layered over 35% (*w*/*w*) sucrose complemented with 10 mM Tris-HCl and 1 mM EDTA (pH 7.4) and centrifuged at 25,000 rpm at 4 °C using a Beckman SW28 swinging bucket rotor in the Beckman Optima LE-80K ultracentrifuge (Beckman Coulter, Brea, CA, USA) for 1 h 10 min. About 8 mL of the opaque interface was recovered in low-sucrose buffer (25 mM sucrose and 10 mM Tris-HCl; pH 7.4) and centrifuged again for 35 min at 25,000 rpm at 4 °C. The supernatant was discarded, while the pellets were resuspended and washed in 1 mL of transport buffer (50 mM Tris-HCl and 250 mM sucrose; pH 7.4) by ultracentrifugation at 55,000 rpm for 20 min using the mini-ultra rotor in the Beckman TL-100 ultracentrifuge. The plasma membrane pellets were resuspended in transport buffer and passed through a 27-gauge needle 20 times for vesicle formation. All centrifuges and rotors were pre-cooled to 4 °C at least 1 h prior to respective centrifugations. The quick-start Bradford Protein Assay kit (BioRad, Hercules, CA, USA) was used to measure protein concentration. 

### 2.5. Two-Color MRP1-Expressing Stable Cell Lines

Stable cell lines expressing two-color MRP1 constructs were prepared by transfecting the two-color MRP1 expression plasmids into HEK293T cells using jetPRIME mammalian transfection reagent (Polyplus-transfection SA, Illkrich, France) following the manufacturer’s guidelines. Cultures were incubated for 24 h before replacing the complete DMEM with medium containing 400 µg/mL Geneticin (G418). After two weeks of maintaining cells, the G418 concertation was doubled to 800 µg/mL. Using flow cytometry, cells expressing both GFP and RFP were sorted from non-expressing cells, and consequently maintained under a lower G418 selection concentration of 200 µg/mL.

### 2.6. Immunoblot Analysis

Lysates of HEK293 cells transfected with two-color and wild-type MRP1 expression vectors were prepared in Halt Protease Inhibitor Cocktail (ThermoFisher Scientific, Waltham, MA, USA) supplemented with radioimmunoprecipitation assay (RIPA) buffer. Pierce bicinchoninic acid (BCA) Protein Assay was the method of choice for protein concentration determination. Prior to an hour-long room-temperature membrane blocking, protein electrophoresis (20 µg of protein) was performed on 7.5% Mini-PROTEAN^®^ TGX™ gels and placed onto an Immobilon^®^ polyvinylidene fluoride (PVDF) membrane (EMD Millipore). After blocking, membranes were incubated at 4 °C overnight using monoclonal anti-MRP1 antibody (IU5C1), or anti-α-tubulin antibody or anti-GFP at 1:250 and 1:5000 dilutions, respectively. Incubation with a secondary antibody was done for an hour at room temperature with horseradish peroxidase-conjugated goat anti-mouse immunoglobulin G (IgG; H+L) and target proteins were detected using enhanced chemiluminescence substrate (PerkinElmer) and the OMEGA LUM G imaging system (Aplegen, Pleasanton, CA, USA). 

### 2.7. Detection of MRP1 Localization 

HEK293T cells were plated and incubated in a humidified 5% CO_2_-supplemented incubator at 37 °C for 24 h before being transfected with the six two-color MRP1 plasmids using jetPRIME transfection reagent (Polyplus-transfection SA, Illkrich, France), following the manufacturer’s protocol. After 48 h of incubation, cells were imaged with a 63× oil objective confocal microscope (TILL Photonics GmbH, Gräfelfing, Germany). GFP and RFP were excited at 470 nm and 561 nm, respectively. Emissions of GFP and RFP were correspondingly achieved at 496–530 nm and 573–637. All images were processed with the ImageJ software (NIH).

### 2.8. Doxorubicin Accumulation Assay

HEK293T cells were plated at 3 × 10^5^ cells/well in 2 mL of complete medium on a coverslip coated with poly-d-lysine in a six-well plate and incubated in a humidified incubator (5% CO_2_, 37 °C). After 24 h, the HEK293T cells were transiently transfected with the six different two-color MRP1 constructs in separate wells using jetPRIME mammalian transfection reagent and incubated as previously for 48 h prior to doxorubicin treatment for 1 h. Images were taken using a confocal microscope equipped with a 63× oil immersion objective and a 1.35 numeral aperture capacity (TILL Photonics GmbH, Gräfelfing, Germany). GFP and Doxorubicin were excited at 470 nm wavelength using Ar laser illumination, with emission bands of 480–530 nm for GFP and 570–605 nm for doxorubicin. RFP was excited at 561 nm with an emission band of 573–637 nm. All images were processed with ImageJ (NIH).

### 2.9. Ensemble Fluorescence Spectroscopy

HEK-293 cells stably expressing GFP–MRP1 or two-color MRP1 were used to prepare membrane vesicles. Steady-state ensemble fluorimetry was used to determine apo and ligand-induced FRET efficiency with minor modifications in the experimental conditions described previously [38]. Briefly, 10 µg of MRP1-enriched membrane vesicles in Tris sucrose buffer (250 mM Tris and 50 mM sucrose; pH 7.4) were kept for 2 min at 37 °C. Membrane vesicles were then incubated with 10 µM of test compounds for 10 min at 37 °C in a water bath prior to FRET measurements. Fluorescence spectroscopy was carried out in a 50-uL quartz glass cuvette with the Fluorimeter model FL3-11. GFP was excited at 465 nm. Emission for GFP was recorded at 480–650 nm with 3 s of integration time. Excitation and emission slit widths of 5 nm were used in all measurements. Firstly, an emission scan of the GFP–MRP1 sample (donor only) was collected while monitoring average donor emission peak. Emission scans were collected for various two-color MRP1 constructs under different experimental conditions (apo or in the presence of a ligand or test compound) while monitoring donor quenching, and the average donor emission peaks were recorded. The emission scan was collected every 3 s with an interval of 5 nm for a total of 10 min. 

To calculate FRET efficiency, the relative fluorescence intensity of the donor in the presence and absence of the acceptor was measured. The ratio of the average donor emission peak intensity in the two-color MRP1 sample to the average donor emission peak intensity in the GFP-MRP1 sample was subtracted from 1 to obtain the transfer efficiency of apo and compound-induced conditions, as shown in Equation (1) [39]. Transfer efficiencies of compound-induced samples were normalized with that of the apo condition to obtain the percent change in FRET.

(1 − *I*_DA_/*I*_D_) × 100, (1)

where *I*_D_ and *I*_DA_ represent the intensities (counts per second, cps) of the donor and acceptor fluorophores, respectively.

### 2.10. Anti-Cancer Drug Screening Using Fluorescence Spectroscopy-Based FRET Approach

To validate the two-color MRP1/FRET model as a viable tool for the discovery of potential modulators of the transporter, 40 anti-cancer drugs were screened using the most sensitive biosensor. Two-color GR-881 was selected as the lead biosensor based on pre-screening results and was, therefore, used for the screening of the 40 clinically tested anti-cancer agents (Appendix A). Firstly, 10 µg of membrane vesicles purified from HEK293/GR-881 were preincubated for 2 min and combined with 10 µM of each of the 40 anti-cancer agents in separate reactions using Tris–sucrose buffer (TSB) as the buffer medium. Prior to FRET measurements with Fluorimeter model FL3-11, the reaction was incubated for 10 min at 37 °C. JNJ-38877605 was used as a negative control for the experiments. Experiments were repeated three times independently, and the percent FRET change was measured and reported as previously described.

## 3. Results and Discussion

### 3.1. Genetic Engineering and Expression of Two-Color MRP1 Recombinant Proteins

**Figure 2:**
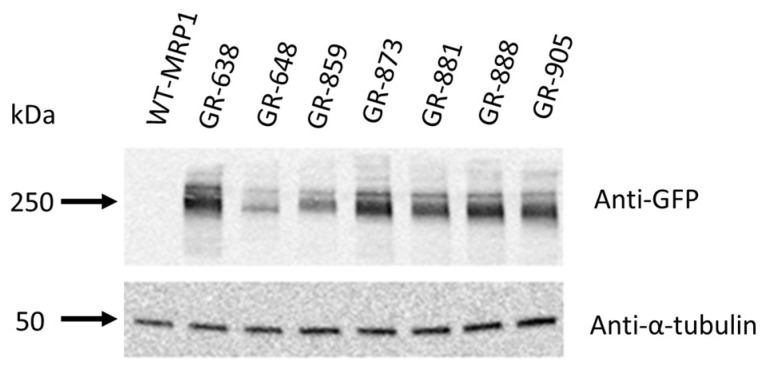
Two-color MRP1 immunoblots. Immunoblots of whole-cell lysates (10 μg) containing wild-type (WT-MRP1) and seven two-color MRP1 constructs. Detection was carried out using rabbit polyclonal anti-GFP antibody (1:5000 dilution, overnight at 4 °C) and mouse monoclonal anti-α-tubulin (1:8000 dilution, overnight at 4 °C).

We previously engineered a two-color MRP1 recombinant protein that could be utilized for high-throughput screening of drug libraries to discover unknown activators, inhibitors, or transportable substrates of MRP1 [26]. In order to make this innovative approach more robust and readily accessible, we decided to create new two-color MRP1 constructs, which are more sensitive to substrate interaction. To test if altering the position of the GFP insertion resulted in enhanced FRET sensitivity, we generated six additional two-color MRP1 constructs by inserting the GFP tag at different sites within the MRP1 coding sequence, while keeping the RFP at the end of MRP1 (Figure 1B). Different GFP insertion sites were chosen using our knowledge of ABC transporter structure–function relationships, guided by the homology models of MRP1 and sequence alignments with related transporters. The other major consideration in the design was to avoid regions critical for the expression and function of MRP1. We chose two sites in the loop region preceding NBD1 and four sites for GFP insertion in the loop region connecting NBD1 to TM12. These regions are predicted to be very flexible and dynamic. Based on available structural information for MRP1 and related ABC drug transporters, the fused GFP and RFP tags are expected not to disrupt the binding of various substrates to the binding site/s formed by the MSDs. MRP1 expression was checked by immunoblot analysis using cell lysates of HEK293 cells transfected with complementary DNA (cDNA) expression vectors encoding wild-type or two-color MRP1, loading 10 µg of whole-cell lysate per lane. The six newly created two-color MRP1 recombinant proteins were GR-638, GR-648, GR-859, GR-881, GR-888, and GR-905. GR-873 two-color MRP1 was the construct made in our previous proof-of-concept study. Tagging MRP1 with GFP and RFP is reflected by GR, and numbers in the name indicate the GFP insertion site. For instance, in GR-638, GFP is inserted after residue 638 of the wild-type MRP1 sequence. Two-color MRP1 proteins were detected using anti-GFP antibody, and the immunoblot results indicated the expected molecular mass of ~250 kDa for all new two-color MRP1 recombinant proteins, similar to the previously characterized protein GR-873, an indication of the collective sizes of the GFP (27 kDa), TagRFP (27 kDa), and the 190-kDa wild-type MRP1 (Figure 2). We demonstrated earlier that the GR-873 expression level and activity are same as wild-type MRP1 (WT-MRP1) protein. Based on immunoblot analysis, expression levels of GR-638, GR-881, GR-888, and GR-905 were normal and similar to GR-873, and, as expected, WT-MRP1 was not detected with anti-GFP antibody. However, the expression levels were moderately reduced for GR-859 and dramatically reduced for GR-648. The insertion of GFP after residue 859 or 648 of MRP1 likely interfered with the proper folding of MRP1 because both insertion sites were closest to NBD1, which is a highly conserved domain in the ABC family of transporters.

### 3.2. Localization and Transport Activity of Two-Color MRP1 Proteins in Live Cells

**Figure 3:**
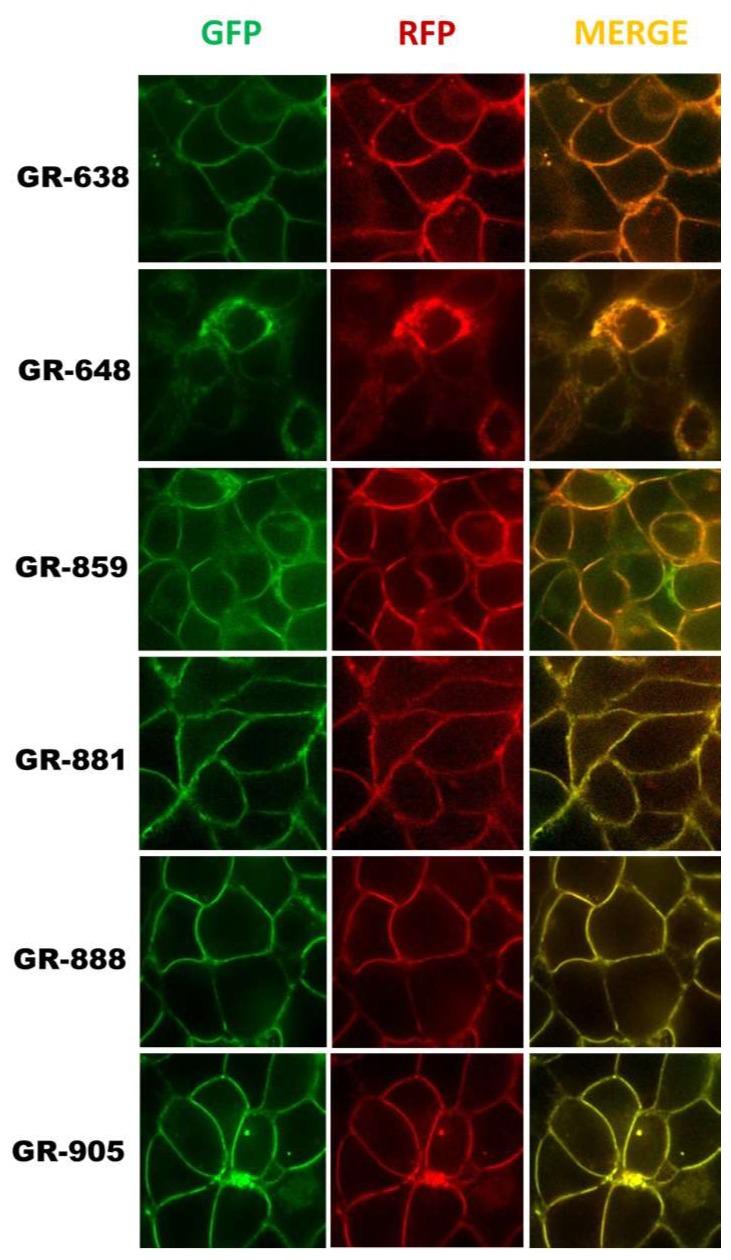
Localization and expression of two-color MRP1 proteins. HEK-293 (human embryonic kidney) cells were plated on glass-bottom chambered coverslips as described in Section 2.8. Fluorescent images were taken using a confocal microscope equipped with a 63× oil-immersion objective. GFP and TagRFP were excited at 470 nm and 561 nm, respectively. Emission was collected at 480–530 nm for GFP and 580–669 nm for RFP.

Fluorescent protein tags like GFP or RFP are usually fused at the amino-terminal or carboxyl-terminal, and do not often cause protein folding and trafficking issues. However, insertion of GFP within the coding sequence of MRP1 could potentially cause problems with the correct folding and trafficking of MRP1. Although all the two-color MRP1 recombinant proteins showed the expected size, we wanted to make sure that both fluorophores (GFP and RFP) matured properly and that the recombinant MRP1 proteins were folded, trafficked, and localized properly at the plasma membrane of cells. HEK293 cells were transfected with cDNA expression vectors encoding different two-color MRP1 proteins, and confocal microscopy was used to visualize the localization and expression levels of recombinant MRP1 proteins. Confocal images in Figure 3 showed that all two-color MRP1 recombinant proteins except GR-648 localized properly at the plasma membrane. In addition, upon acquiring images using the GFP and RFP channel, and analyzing the merged images, it was obvious that both fluorophores matured properly. However, insertion of GFP in the GR-648 recombinant protein was not tolerated and induced misfolding and processing defects, leading to reduced levels of the recombinant protein, as well as intracellular retention; therefore, this construct was not included in further investigation. The GR-859 recombinant protein showed partial mislocalization and intracellular retention that can be attributed to the moderately reduced levels observed in the Western blots, but the recombinant protein was predominantly localized at the plasma membrane. 

**Figure 4:**
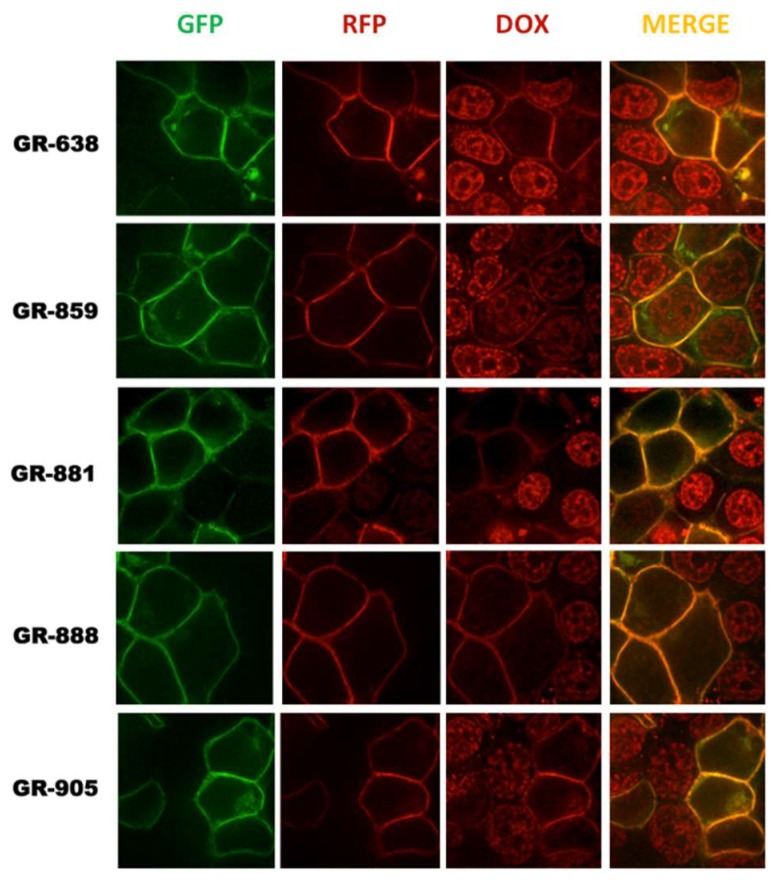
Doxorubicin (Dox) accumulation assay. HEK293T cells were transiently transfected with six different two-color MRP1 constructs and incubated for 48 h, after which doxorubicin treatment was done. Images were taken using a confocal microscope equipped with a 63× objective. GFP and Dox were excited at 470 nm wavelength using an Ar laser, with emission bands of 480–530 nm for GFP and 573–637 nm for Dox. RFP was excited at 561 nm, and its emission was collected at 580–669 nm. Data were collected from all three channels—GFP, RFP and Dox. RFP and Dox have significant emission wavelength overlap, explaining the membranous signal seen for the two-color MRP1 recombinant proteins in the Dox channel.

To determine if the engineered two-color MRP1 recombinant proteins were functional, their transport activities were evaluated in live cells by measuring accumulation of the fluorescent anti-cancer drug, doxorubicin, a well-known substrate of MRP1 [40,41]. HEK293 cells were transfected with cDNA expression vectors encoding different two-color MRP1 proteins, and confocal microscopy was used to visualize the accumulation of doxorubicin (Dox) inside the cells. Images were captured for GFP, RFP, and Dox, and images were merged for analysis. Transiently transfected cells are expected to have a mixed population of cells, whereby some cells that pick up the vector DNA will express two-color MRP1 while untransfected cells will not at the plasma membrane. HEK293 cells are known to express extremely low or negligible levels of endogenous MRP1. Confocal microscopy of transiently transfected HEK293 cells showed high doxorubicin accumulation in the nucleus of untransfected cells, but doxorubicin fluorescence was very low or negligible in cells expressing either GR-638, GR-881, GR-888, or GR-905 (Figure 4). These results demonstrate that recombinant MRP1 proteins GR-638, GR-881, GR-888, and GR-905 were functionally active. In contrast, HEK293 cells transfected with GR-859 showed high doxorubicin accumulation in the nucleus, indicating that this recombinant protein was not functional, despite having the expected size and proper localization at the plasma membrane. Consequently, GR-859 was not included in further studies.

### 3.3. Substrate-Free FRET Efficiencies of the Two-Color MRP1 Proteins 

**Figure 5:**
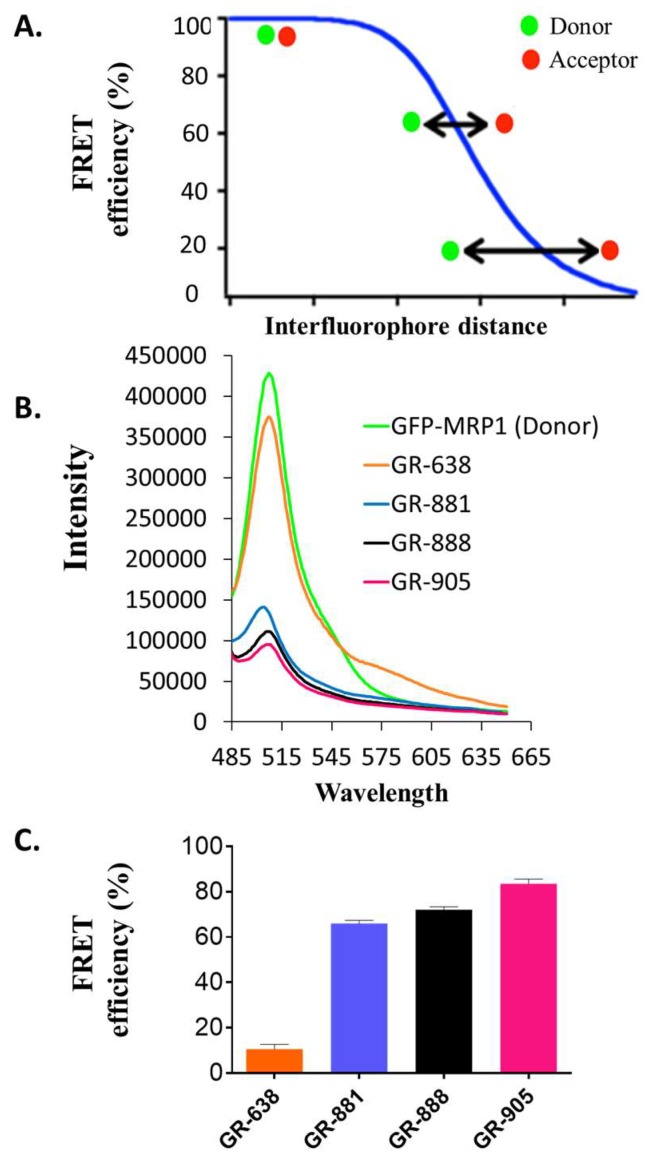
Ligand-free (apo) fluorescence resonance energy transfer (FRET) efficiencies of the two-color MRP1 constructs. (**A**) Graphical representation of the influence of inter-fluorophore distance on FRET efficiency. The more proximal the fluorophores are, the higher the FRET efficiency. The higher the apo FRET is, the higher the change in transfer efficiency, until a certain threshold is reached. (**B**) Fluorescence spectra of different two-color MRP1 proteins (GFP–MRP1 used as donor control). (**C**) Ligand-free FRET efficiency of different two-color MRP1 proteins. Membrane vesicles were prepared from HEK-293 cells stably expressing different two-color MRP1 proteins (GR-638, GR-881, GR-888, and GR-905). Firstly, 20 µg of each two-color protein in Tris–sucrose buffer was prepared and measured for ligand-free FRET with Fluorimeter model FL3-11 using a 465-nm excitation wavelength and 480–650 nm as the emission wavelength range for GFP. Three independent experiments were done using two different membrane vesicle batches. Intensities of the two-color MRP1 constructs were normalized to the donor GFP–MRP1. FRET efficiency was calculated using Equation (1).

FRET is a very useful tool to detect biochemical interactions, such as protein–protein interactions and protein–DNA interactions, and to study protein structural dynamics [26,42,43,44,45,46,47,48]. The efficiency of this nonradiative energy transfer is inversely proportional to the sixth power of the distance between donor and acceptor fluorophores, which makes the FRET approach extremely powerful for detecting conformational changes within a protein. Figure 5A shows a graphical representation of the relationship between FRET efficiency and the distance between the donor and acceptor fluorophores. Our goal was to create a two-color MRP1 biosensor that produces a high FRET efficiency change upon substrate interaction. If the intramolecular FRET efficiency of the apo (FRET in the absence of substrate) two-color MRP1 is too low, then the FRET biosensor protein would not be very sensitive reporter to small distance changes in the structure of the protein (representing the bottom part of the curve in Figure 5A). Similarly, if the intramolecular FRET efficiency of the apo two-color MRP1 is too high, then the FRET biosensor protein would also not be very sensitive to small distance changes in the structure of the protein (representing the top part of the curve in Figure 5A). A two-color MRP1 protein that exhibits an intramolecular FRET efficiency in the range of approximately 30–70% is desirable, because the curve is the steepest in that region; correspondingly, a small structural change upon substrate binding can result in a higher FRET change from the basal level [49].

Previously, we engineered a two-color MRP1 protein (GR-873) that reported substrate-induced intramolecular FRET change as a function of structural changes in the nucleotide domains of MRP1 [26]. To determine the basal intramolecular FRET efficiencies of the new two-color MRP1 proteins, we employed steady-state fluorescence spectroscopy using a fluorometer. To keep the protein in its native environment, membrane vesicles isolated from HEK293 cells stably expressing different two-color MRP1 proteins were used for the FRET experiments. As shown in Figure 5B, fluorescence intensity of the donor (GFP) was highest for the GFP-MRP1 construct (donor-only construct with no RFP). The fluorescence intensity of GFP decreased to varying degrees in the different two-color MRP1 recombinant proteins due to donor quenching in the presence of the acceptor, RFP. The shorter the inter-fluorophore distance between the inserted GFP and the terminal RFP, the higher the apo FRET efficiency of the construct. The calculated intramolecular FRET efficiency (%) for GR-638, GR-881, GR-888, and GR-905 was 10.5 ± 1.5%, 66.0 ± 1.0%, 72 ± 1.0%, and 83.5 ± 1.5%, respectively (Figure 5C). This indicates that the distance between GFP and RFP is shortest in GR-905 and longest in GR-638. The intramolecular FRET efficiency (%) for apo GR-873 was previously reported to be ~16%. Based on these data, GR-881, GR-888, and GR-905 appear very promising in terms of reporting higher FRET efficiency changes upon interaction with substrate.

### 3.4. Evaluation of Two-Color MRP1 Proteins as FRET-Based Biosensors 

**Figure 6:**
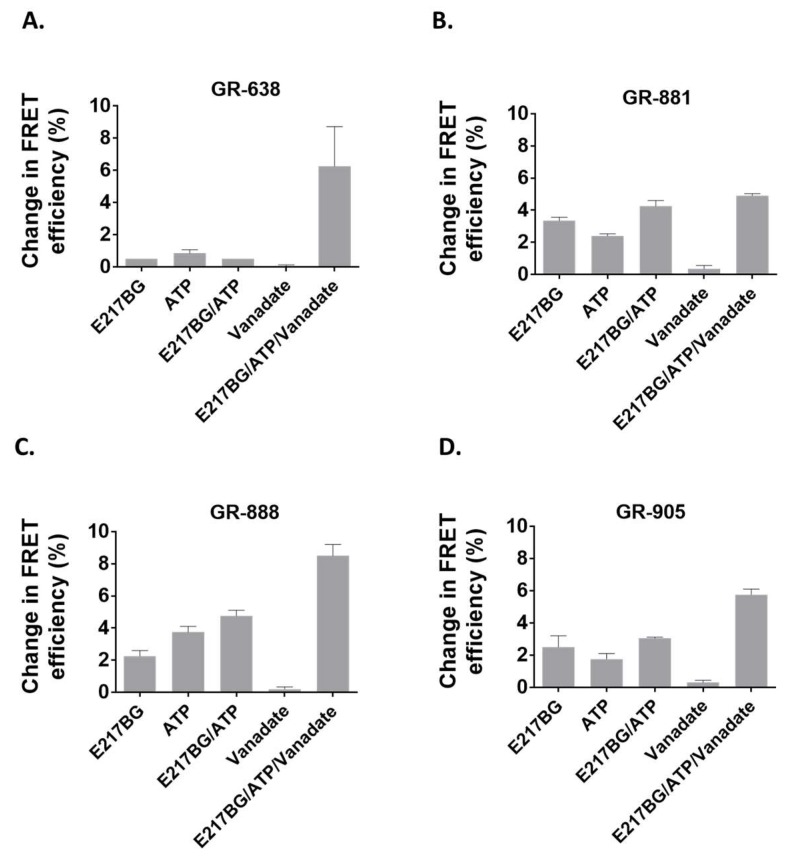
Ligand-dependent intramolecular FRET measurements. (**A**) Ligand-dependent FRET changes of GR-638 protein. (**B**) Ligand-dependent FRET changes of GR-881 protein. (**C**) Ligand-dependent FRET changes of GR-888 protein. (**D**) Ligand-dependent FRET changes of GR-905 protein. Firstly, 20 µg of protein in Tris–sucrose buffer was prepared and incubated with 5 µM estradiol glucuronide (E217βG) and/or 4 mM/5 mM ATP/MgCl_2_ and 1 mM sodium vanadate for 10 min prior to FRET measurements using the fluorimeter. GFP excitation was done at 465 nm, and emission was collected at 480–650 nm. The results shown are means ± SD of triplicate measurements in a single experiment. Similar results were observed in a second independent experiment done in triplicate using a different membrane vesicle batch.

To determine the capacity of the engineered two-color MRP1 proteins as biosensors and reporters of conformational changes in the NBDs upon substrate binding, we measured ligand-dependent structural changes of the MRP1 protein through steady-state FRET spectroscopy using the fluorometer. Membrane vesicles isolated from HEK293 cells stably expressing different two-color MRP1 proteins were used for the FRET experiments. In the presence of E217βG (a well-known physiological substrate of MRP1) [50,51,52], the average FRET efficiency changes observed for GR-638, GR-881, GR-888, and GR-905 were 0.5, 3.35, 2.25, and 2.5%, respectively (Figure 6). These results are in agreement with earlier findings that reported conformational changes in the NBDs upon interaction with substrate alone [29,38]. Different two-color MRP1 proteins also showed a range of FRET efficiency changes in the presence of ATP alone or E217βG + ATP. With the exception of GR-638, all the two-color MRP1 proteins produced higher FRET efficiency changes in the presence of E217βG + ATP compared with either E217βG or ATP alone. This is expected because the presence of both E217βG and ATP should shift the equilibrium toward the closed conformation of the transporter. In addition, all two-color MRP1 proteins produced the highest FRET efficiency change when the transporter was trapped in the closed conformation (E217βG + ATP + vanadate) and this is consistent with the vanadate-locking phenomenon reported for other ABC transporters [38,53]. It is important to note that our previously engineered GR-873 was not able to show detectable FRET change in the presence of E217βG alone, whereas GR-881, GR-888, and GR-905 exhibited significant FRET efficiency changes in the presence of E217βG alone. As shown in Figure 6, the three two-color MRP1 proteins (GR-881, GR-888, and GR-905) consistently produced higher FRET efficiency changes in various tested conditions, while the highest FRET efficiency change was observed with GR-881 upon interaction with the substrate alone.

### 3.5. Identification of Anti-Cancer Drugs that Interact with MRP1

**Figure 7:**
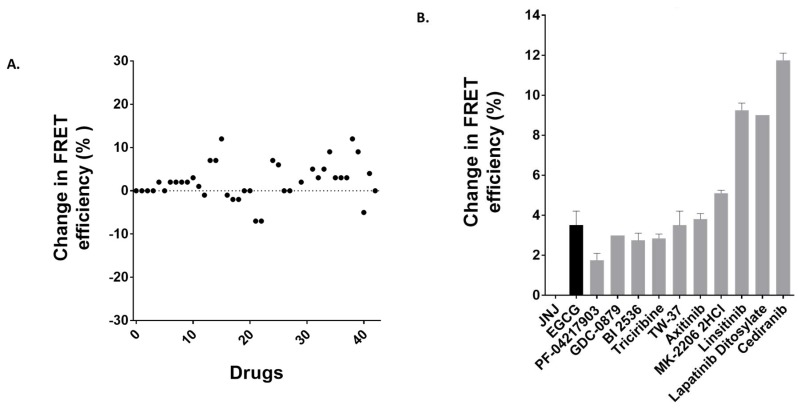
Anti-cancer drug screening with two-color GR-881. (**A**) Change in FRET efficiency (%) for the 40 anti-cancer drugs from one trial. (**B**) Ten anti-cancer compound hits which increased FRET and produced high and consistent FRET changes in three independent screening experiments using two different membrane vesicle batches. FRET measurements were performed using Fluorimeter model FL3-11. Donor was excited at 465 nm, and emission was collected within the 480–650 nm range and donor quenching was observed. Data are represented as means ± standard error of the mean (SEM) from three independent experiments. Anti-cancer drug JNJ 38877605 is included as a negative control.

**Figure 8:**
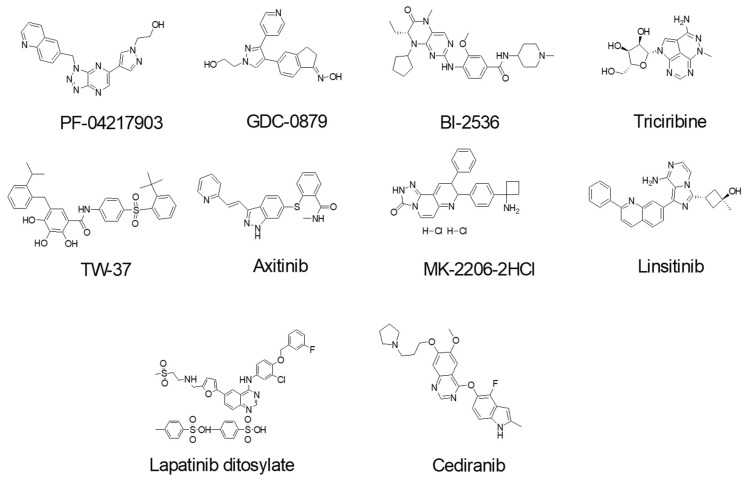
Chemical structures of the ten anti-cancer drug hits.

To further validate the two-color MRP1 protein model as a potent biosensor system, and two-color MRP1-coupled steady-state fluorescence spectroscopy as a viable tool for the drug screening to identify novel substrates, inhibitors, or activators of MRP1, we screened 40-novel clinically tested anti-cancer drugs. GR-881 was chosen for this screening because it was found to be most sensitive to substrate binding. Membrane vesicles isolated from HEK293 cells stably expressing the GR-881 MRP1 biosensor were used in the fluorescence spectroscopy-based FRET experiments. We screened the 40-drug library at least two independent times for their interaction with the GR-881 MRP1 biosensor. Results from a single screening are presented in Figure 7A. Ten drugs were identified (structures shown in Figure 8) which increased FRET and produced high and consistent FRET changes in three independent experiments (Figure 7B). Means of the FRET efficiency changes observed for the identified hits were as follows: PF-04217903 (1.75%), GDC-0879 (3%), BI-2536 (2.75%), triciribine (2.85%), TW-37 (3.5%), axitinib (3.8%), MK-2206 (5.1%), linsitinib (9.25%), lapatinib ditosylate (9%), and cediranib (11.75%) (Figure 7B). Epigallocatechin gallate (EGCG), a known substrate of MRP1 and one of eight compounds previously identified in the FRET-based screening, was used as a positive control and produced a mean value of 3.5% FRET efficiency change. JNJ-38877605, an anti-cancer drug which does not interact with MRP1, was used as a negative control, and no detectable FRET efficiency change was observed in this case. 

In the present study, using our newly engineered GR-881 MRP1 biosensor, we identified 10 compounds that are suggested to directly interact with and induce a conformational change in the structure of MRP1 after screening only 40 anti-cancer drugs. The identified compounds from this screen could be substrates, inhibitors, activators, or molecules that simply interact with MRP1 to stimulate transport of a substrate or bind with the transporter for unknown reasons, or could be a false hit due to non-specific interaction with MRP1. Preliminary data in a related ongoing project suggest that three of these 10 hits (triciribine, MK-2206, and cediranib) interact with MRP1, and we plan on conducting a detailed investigation for all 10 hits to understand and confirm the biochemical nature of their interaction with MRP1. Our results indicate that the GR-881 MRP1 biosensor is a very powerful tool for identifying drugs and compounds that interact with MRP1. 

## 4. Conclusions

We previously engineered a two-color MRP1 protein and demonstrated the proof of concept of measuring intramolecular FRET efficiency as an index of protein conformational changes upon ligand binding. When this recombinant protein was used in screening a library of 446 drug-like compounds, we identified eight hit compounds (~2% of total compounds screened). In the present study, we engineered six additional two-color MRP1 recombinant proteins and, after detailed characterization, identified three two-color MRP1 proteins (GR-881, GR-888, and GR-905), which report improved substrate-induced intermolecular FRET changes as a function of structural changes of the protein. The two-color MRP1 biosensor GR-881 was used to screen 40 anti-cancer drugs, and was able to identify 10 drugs as hits (25% of total compounds screened). The newly engineered GR-881 has an apo FRET of 66% as compared to 16% apo FRET for the previous construct. Based on Figure 5A, GR-881 falls in the middle region of the sigmoidal curve and is expected to produce a higher FRET signal upon a certain structural change. In addition, we think the use of MRP1-enriched membrane vesicles as an experimental matrix instead of cells reduces the complexity of reaction matrices and avoids nonspecific noise, thereby enhancing the overall intramolecular FRET sensitivity in the steady-state fluorescence spectroscopy-based approach. 

Traditionally, researchers focused on functional-based approaches to identify modulators of ABC transporter proteins. For MRP1, the membrane-vesicle-based in vitro radiolabeled E217βG uptake assay is commonly used to identify modulators [54,55]. However, MRP1 and other drug transporters are expected to have multiple distinct substrate-binding sites. A functional assay based on quantifying transport is unable to detect substrates that bind to a different site and fail to compete with the test substrate. Currently, there is no established high-throughput assay available to identify substrates of MRP1. The idea of the intramolecular FRET-based MRP1 biosensor approach to identifying substrates and modulators is highly innovative and transformative. This approach could potentially be applied to other efflux transporters, and especially to ABC proteins with no known substrate or functional assay. In addition to the discovery of novel substrates and modulators, the two-color MRP1 biosensor protein could be used for single-molecule FRET-based studies to understand the structural dynamics of MRP1 in the native membrane environment. Future projects will focus on the development of a vesicular transport coupled LC–MS/MS assay to verify the MRP1 substrate status of the hits. We also plan on developing a two-color MRP1 fluorescent plate-reader-based high-throughput screening assay to identify more novel substrates of MRP1.

## Appendix A. List of the 40 Anti-Cancer Drugs for FRET Screening with GR-881

ABT-263	Navitoclax
Afatinib	BIBW2992
PD0325901	
Trichostatin A	TSA
BMS-599626	AC480
AUY922	NVP-AUY922
Brivanib	BMS-540215
PF-04217903	
BI 2536	
TW-37	
Mocetinostat	MGCD0103
SRT1720	
YM155	
MLN8237	Alisertib
AT9283	
Andarine	GTX-007
AZD6244	Selumetinib
CI-1040	PD184352
Motesanib Diphosphate	AMG-706
Tandutinib	MLN518
Entinostat	MS-275 SNDX-275
SB 431542	
SU11274	
KU-55933	
LY294002	
XL147	
Saracatinib	AZD0530
Dovitinib	TKI-258
Lenalidomide	Revlimid
Sunitinib Malate	Sutent
Elesclomol	
GDC-0941	
MK-2206 2HCl	
Linsitinib	OSI-906
GDC-0879	
Triciribine	Triciribine phosphate
Axitinib	
Cediranib	AZD2171
Lapatinib Ditosylate	Tykerb
STF-62247	

## Appendix B. Primers used in This Study

**Primer Names**	**Sequences (5’ → 3’)**
MRP1_1–638_ forward	GTA GAG CTC ATG GCG CTC CGG GGC TTC TGC AG
MRP1_1–638_ reverse	CTA GTC GAC ATT CCT CAC GGT GAT GCT GTT CGT GCC C
MRP1_639–1531_ forward	GTA CCG CGG GCC ACA TTC ACC TGG GCC AGG AGC
MRP1_639–1531_ reverse	GTA ACC GGT CT CAC CAA GCC GGC GTC TTT GGC CAT G
MRP1_1–648_ forward	GTA GAG CTC ATG GCG CTC CGG GGC TTC TGC AG
MRP1_1–648_ reverse	CTA GTC GAC ATT CCT CAC GGT GAT GCT GTT CGT GCC C
MRP1_649–1531_ forward	GTA CCG CGG GCC ACA TTC ACC TGG GCC AGG AGC
MRP1_649–1531_ reverse	GTA ACC GGT CT CAC CAA GCC GGC GTC TTT GGC CAT G
MRP1_1–859_ forward	GTA GAG CTC ATG GCG CTC CGG GGC TTC TGC AG
MRP1_1–859_ reverse	CTA GTC GAC GTC TCG AGC CAG CAG CTC CTG GTA GG
MRP1_860–1531_ forward	GTA CCG CGG GGC GCC TTC GCT GAG TTC CTG
MRP1_860–1531_ reverse	GTA ACC GGT CT CAC CAA GCC GGC GTC TTT GGC CAT G
MRP1_1–881_ forward	GTA GAG CTC ATG GCG CTC CGG GGC TTC TGC AG
MRP1_1–881_ reverse	CTA GTC GAC GTT CTC CTC TGC ATC CTG CTC CTG C
MRP1_882–1531_ forward	GTA CCG CGG GGG GTC ACG GGC GTC AGC GGT
MRP1_882–1531_ reverse	GTA ACC GGT CT CAC CAA GCC GGC GTC TTT GGC CAT G
MRP1_1–888_ forward	GTA GAG CTC ATG GCG CTC CGG GGC TTC TGC AG
MRP1_1–888_ reverse	CTA GTC GAC ACC GCT GAC GCC CGT GAC CCC GT
MRP1_889–1531_ forward	GTA CCG CGG CCA GGG AAG GAA GCA AAG CAA ATG GAG
MRP1_889–1531_ reverse	GTA ACC GGT CT CAC CAA GCC GGC GTC TTT GGC CAT G
MRP1_1–905_ forward	GTA GAG CTC ATG GCG CTC CGG GGC TTC TGC AG
MRP1_1–905_ reverse	CTA GTC GAC ACT GTC CGT CAC CAG CAT GCC ATT CTC C
MRP1_906–1531_ forward	GTA CCG CGG GCA GGG AAG CAA CTG CAG AGA CAG C
MRP1_906–1531_ reverse	GTA ACC GGT CT CAC CAA GCC GGC GTC TTT GGC CAT G
GFP-forward	GTA GTC GAC ATG GTG AGC AAG GGC GAG GAG CTG
GFP-reverse	CTA CCG CGG CTT GTA CAG CTC GTC CAT GCC GAG AG
